# Facilitating equity-oriented cancer care in organizations delivering cancer services: insights from a qualitative study

**DOI:** 10.3389/fonc.2026.1676594

**Published:** 2026-04-21

**Authors:** Tara C. Horrill, Jess Crawford, Scott M. Beck, Amber Bourgeois, Jagbir Kaur, Leah K. Lambert, Michael McKenzie, Kelli I. Stajduhar, Annette J. Browne

**Affiliations:** 1 College of Nursing, University of Manitoba, Winnipeg, MB, Canada; 2 School of Nursing, University of Victoria, Victoria, BC, Canada; 3 BC Cancer, part of the Provincial Health Services Authority, Victoria, BC, Canada; 4 BC Cancer Research Institute, Vancouver, BC, Canada; 5 School of Nursing, University of British Columbia, Vancouver, BC, Canada

**Keywords:** cancer care facilities, equity-oriented care, health equity, health services accessibility, patient-centered care

## Abstract

**Background:**

People experiencing structural marginalization often have advanced cancer diagnoses and higher cancer-related mortality resulting, in part, from poor access to care. A key pathway to addressing health outcome disparities includes integrating equity-oriented approaches to cancer care (EOCC). Implementing equity-oriented approaches can help address access to care and quality of care gaps, however little is known about the organizational factors required to support the delivery of EOCC; our study addressed this gap.

**Methods:**

We employed a critical ethnographic approach, guided by critical and intersectional theories, and participatory knowledge translation approaches. We conducted interviews with health and social service providers and key informants, observations of service providers in oncology settings, and interviews and focus groups with people experiencing structural marginalization. Data analysis followed an interpretive descriptive approach, informed by critical ethnographic methodology and critical and intersectional theories.

**Results:**

Our findings point to three dynamic and intertwined dimensions within organizations delivering cancer services that support an equity-oriented approach to care. First, *identifying core values for creating an equity-oriented culture* included values of relationality, cultural safety, adaptability, and trust-building as foundational for EOCC. Second, *building a shared commitment to EOCC* meant dedicated resources and investments, such as time, money, leadership, and partnerships. Finally, *taking action towards EOCC* underscores the importance of accountability and highlights tangible strategies for organizations to enact EOCC.

**Conclusions:**

Cancer care organizations are increasingly recognized as critical sites for advancing health equity. Our findings highlight the important role of shared organizational values and formalized commitments to equity, providing direction for integrating EOCC at the organizational level. Findings also emphasize the importance of attending to organizational contexts as key in supporting clinicians to provide tailored, person-centred, and culturally safer care.

## Introduction

In the Canadian cancer care sector, the last several decades have seen a growing emphasis on advancing health equity, including equitable cancer outcomes and access to cancer care. This is in response to the emerging body of research demonstrating that groups experiencing structural marginalization^1^ are significantly more likely to be underrepresented in cancer control programs and to be diagnosed with cancers that are preventable and/or at advanced stages, and less likely to receive adequate treatment after their cancer diagnosis (1–5). There is compelling evidence that people experiencing structural marginalization (for example, people experiencing poverty, unstable housing, racialization, and other intersecting forms of oppression) are not well-served by organizations delivering cancer services, pointing toward issues with healthcare quality and equity. Our research has highlighted how cancer care organizations in Canada are in an evolving process of fostering health equity through improvements in care delivery approaches and revising institutional policies and processes. These improvement efforts stem from growing awareness that the cancer care sector often lacks capacity to meet the intersecting health and social needs of those most impacted by inequities (6–10).

In Canada, the majority of cancer services are publicly funded, organized provincially, and delivered in a range of settings across the cancer continuum, including in primary, acute and community care settings, and through dedicated provincial cancer agencies (11). Organizations delivering cancer services are increasingly recognized as key sites for health equity initiatives, given the inequitable distribution of cancer risk, outcomes and mortality among populations experiencing structural marginalization (12), and the factors that create and sustain these inequities (13, 14). Efforts to address inequities in access to cancer care and improve cancer outcomes among people experiencing structural marginalization must include the development of organizational capacity for health equity action and organizational structures to support the integration of equity-oriented approaches into the design and delivery of care (15–17). Browne and colleagues (16, 18) have conceptualized equity-oriented healthcare (EOHC) as an approach to improving care that focuses on reducing the impacts of structural inequities (e.g., poverty, discrimination), and mismatches between existing approaches to care and the actual needs of people experiencing structural marginalization by informing organizational-level changes in how services are designed and delivered. EOHC is founded on three key dimensions: 1) trauma- and violence-informed care, 2) culturally safe and anti-racist care, and 3) harm reduction and destigmatizing approaches toward substance use (16, 18). Although, to date, research examining the effectiveness of EOHC has focused primarily on primary care and emergency department settings (19–22), these approaches are relevant to cancer care (15), and care grounded in cultural safety, trauma- and violence-informed approaches, and harm reduction philosophies is critical to improving access to cancer care and patient experiences of care (9, 21, 24). Recent research within the cancer care sector suggests that, although healthcare providers play a vital role in delivering equity-oriented *cancer* care (EOCC), their capacity to do so is enabled or limited by the organizational contexts and health systems in which they work (e.g., the structure of healthcare providers’ work; the bureaucratic and siloed nature of the healthcare system) (9, 10). Organizational culture and values are increasingly being understood as critical to advancing equity and implementing health equity interventions (16, 17, 25, 26). However, recent evidence suggests that data to inform how to build that culture are limited (27); moreover, there is little evidence on how to create an organizational environment that can support the shifts needed to foster greater equity within cancer care. The purpose of this study was to examine the integration of equity-oriented care approaches into cancer care services in one Western Canadian province. This paper reports on our analysis of organizational factors that facilitate an equity-oriented approach to cancer care; reporting follows the SRQR guidelines (28) and COREQ extension guidelines (29).

## Methods

This study drew on critical ethnographic methods (30, 31), and participatory and integrated knowledge translation approaches (32, 33). As such, healthcare providers and leaders working within the cancer care sector were a part of the research team and involved in all aspects of the study. As part of the study, we partnered with a community-based primary care clinic who have a mandate to provide health and social services to people who are structurally marginalized in one large urban area.

### Theoretical & methodological approaches

We approached the study through a constructivist epistemological lens, which acknowledges reality as constituted through social interactions and experiences while emphasizing the role of contexts in creating meaning. Grounded in social justice aims, we drew on critical and intersectional theoretical perspectives as we designed and conducted the study (34, 35). Critical perspectives theorize that social identities like age, race, class and gender, as well as social institutions like economic, cultural, and political institutions intersect and interact to construct systems of oppression and individual experiences (36, 37). These theories focus our attention on how complex social, political, and power dynamics impact the design and delivery of healthcare services and the ability of healthcare providers to meet the needs of patients. Intersectional theories focus on how various social identities interact to construct experiences of privilege and oppression; these theories have been applied in health research to examine how various intersecting identities create differential experiences of health and healthcare, and differing access to care (38, 39). In line with critical and intersectional theories, critical ethnographic methods aim to investigate issues of health and social justice, advance equity stimulate social change (40). Critical ethnographic methods are ideally suited to studying organizational contexts, and focused our inquiry on the development of knowledge to effect change and address inequities in the delivery of cancer services. Interpretive description (ID) informed our approach to data analysis (41). For readers less familiar with ID, the goal of analysis is not to generate generalizable knowledge claims, but insights beyond the self-evident, and applicable to clinical contexts while acknowledging the constructed and contextual nature of knowledge; as such, an ID approach aligned well with our theoretical and methodological orientations (42).

### Research team & positionality

Our research team included academic researchers, clinician-scientists, and direct care providers, all of whom have professional healthcare designations (RN, NP or MD). Our team brings clinical and research experience in cancer and primary care contexts. The team was co-led by TH, AJB and KS. Several team members worked within the oncology settings from which data were collected (SB, AB, JK, LKL and MM); AJB has a long-standing research relationship with the community-based primary care clinic that we partnered with which supported further relationship building and reciprocal knowledge exchange.

### Data collection

This study was conducted in two large urban areas of one Western Canadian province, with data collection occurring between June 2022 and December 2023. Purposive and nominated sampling techniques were used to recruit: (a) health and social service providers (e.g., nurses, nurse practitioners, physicians) working in an oncology setting or in a community-based setting who had experience providing care to structurally marginalized people (‘service providers’; n=24, **Table 1**); and (b) organizational, clinical, or policy leaders working across the cancer continuum (‘key informants’; n=7, **Table 2**). Invitations to participate were sent to service providers and key informants by email through the community-based primary care clinic we partnered with, as well as through our professional networks; study information was also posted online. Participating service providers could also refer potential participants through nominated (“snowball”) sampling. For ethical and pragmatic reasons, convenience sampling was used to invite a third group: people with lived/living experience of structural marginalization (e.g., poverty, unstable housing, food insecurity) (‘community members’; n=29, **Table 3**). In recognition of the reality that many people experiencing structural marginalization may never receive an official cancer diagnosis or receive cancer treatment (as a result of the intersecting barriers to care), we did not require community participants to have a confirmed cancer diagnosis to participate (although many disclosed personal experiences across the cancer continuum and/or experiences of cancer care through their support of a loved one with cancer). To invite this group, we posted invitations to participate in the facility operated by our partner organization. Study information was also provided to community members who met inclusion criteria through community-based health and social service providers in our partner organization.

**Table 1:** Service provider participants*.

Participant characteristics	N=24 (%)
Employment Role	
Nurse (RN)	7 (29%)
Nurse Practitioner	6 (25%)
Physician	3 (13%)
Outreach/Support Worker	2 (8%)
Allied Health Professional	2 (8%)
Manager/Coordinator	2 (8%)
Other	2 (8%)
Setting	
Community-based or primary care	14 (58%)
Oncology care	9 (38%)
Years in Current Role	
1-5	13 (54%)
6-10	3 (13%)
11-15	5 (13%)
16-20	2 (8%)
20+	1 (4%)

*Eligibility criteria: Health or social service provider; working in urban community-based settings with structurally marginalized populations OR cancer care sector; provide or have provided direct care to structurally marginalized adults with suspected or confirmed cancer; >18 years old; able to speak English.

**Table 2:** Key informant participants** .

Participant characteristics	N=7 (%)
Employment Role	
Executive or operational leader	2 (29%)
Clinical leader	3 (42%)
Policy maker	2 (29%)
Setting	
Community-based or primary care	1 (14%)
Oncology care	6 (86%)
Years in Current Role	
	0 (0%)
1-5	4 (57%)
6-10	2 (29%)
20+	1 (14%)

**Eligibility criteria: Health systems leaders, administrators, decision makers and/or those considered experts within the cancer care sector; knowledge and experience of factors currently or historically shaping design and delivery of cancer services including attempts to integrate health equity principles into cancer care; >18 years old; able to speak English.

**Table 3:** Community participants***.

Participant characteristics^	N=29 (%)
Age	
Average age	56
Age range	27-77
Gender	
Woman	21 (73%)
Transwoman	1 (3%)
Man	5 (17%)
Not specified	2 (7%)
Indigenous Status	
Self-identified as Indigenous	17 (59%)
Self-identified as non-Indigenous	10 (34%)
Prefer not to answer or not specified	2 (7%)
Highest Level of Education	
Graduate degree	1 (3%)
University degree or college diploma	3 (10%)
Some university or college	3 (10%)
Highschool degree or GED	7 (24%)
Some high school or middle school	10 (34%)
Completed elementary school	2 (7%)
Don’t know or not specified	3 (10%)
Current Living Situation	
Private apartment/condo/house	7 (24%)
Public, social or supportive housing	13 (45%)
Couch surfing	1 (3%)
Shelter	3 (11%)
On the street	1 (3%)
Single-room occupancy hotel	2 (7%)
Prefer not to answer or not specified	2 (7%)
Current Work Status	
Employed full time (20+ hours/week)	1 (3%)
Employed part time (<20 hours/week)	3 (10%)
Seasonally employed	1 (3%)
Unemployed	10 (34%)
Retired	3 (10%)
Services in exchange for food/housing	1 (3%)
Student	2 (7%)
Other	5 (17%)
Prefer not to answer or not specified	3 (10%)
Sources of Income	
Social assistance	4 (14%)
Disability benefits	16 (55%)
Pension	6 (21%)
Prefer not to answer or not specified	3 (10%)
Difficulty Living on Current Household Income	
Very difficult	9 (31%)
Somewhat difficult	13 (45%)
Neutral	1 (3%)
Somewhat easy	4 (14%)
Very easy	0 (0%)
Prefer not to answer or not specified	2 (7%)

***Eligibility criteria: patients who experience significant socioeconomic disadvantage, including poverty, mental health challenges, substance use, absence of a fixed address or telephone, and/or lacking appropriate health insurance; >18 years old; able to speak English.

^Options where n=0 are not reported.

### Focus groups and semi-structured interviews

We conducted semi-structured interviews (audio-recorded) with service providers and key informants in person or via Zoom, according to participant preference. In-person focus groups with community members were conducted between October and December of 2023; several additional semi-structured virtual interviews were conducted with community members who wanted to participate but were unable to attend a focus group. A cash honorarium was offered to community member participants, and to service providers who participated outside of their regular work responsibilities. Interviews and focus groups were led by the first author, and ranged from 20–90 minutes for interviews, and 60–120 minutes for focus groups. Following semi-structured interview guides, interviews with service providers focused on understanding how their organizations supported the provision of EOCC and perspectives on how to shift cancer service delivery to be more equitable and accessible; interviews with key informants focused on organizational strategies to integrate health equity approaches and the organizational factors that support or constrain them. Focus groups took a strengths-based approach to understand how to make people experiencing structural marginalization feel as comfortable as possible when accessing cancer services, and how to improve experiences of care. Interviews were professionally transcribed; detailed fieldnotes were recorded by hand during focus group discussions and transcribed afterwards.

### Observations

An equity-oriented approach (43) was used to conduct observations in two urban cancer centers between October 2022 and October. The first author spent approximately 40 hours conducting observations of service providers working in clinical oncology settings (e.g., outpatient oncology clinics, radiation treatment clinics), however, the primary focus of observations was not the providers themselves, but the organizational contexts of care delivery that structured their capacity to deliver equity-oriented care. Additional details of observations are reported elsewhere (10) Field notes were used to record observations (e.g., physical environment and layout, visible signage, service provider interactions, patient flow processes) alongside reflexive (e.g., personal reflections of the researcher), analytical (e.g., how observations reinforced or diverged from interview data, how observations aligned with the theoretical frameworks), and methodological notes (e.g., possible modifications to interview guide).

### Data analysis

Interview transcripts, focus group notes, and observational field notes were imported into Dedoose qualitative analysis software (www.dedoose.com) to support data management and retrieval. We applied an ID approach to data analysis (41), and data were analyzed inductively and iteratively. Coding was conducted by two team members (TH and JC), beginning with repeated readings of interview transcripts and field notes and the development of an initial broad coding framework; this framework was refined, revised and applied by JC as data analysis progressed (44). As patterns emerged, we grouped coded data into categories, using visual and memoing strategies to diagram the relationships between codes and categories. Throughout these stages, TH and JC met bi-weekly to discuss analytic insights, with specific attention to ensuring our analysis was informed by critical theoretical perspectives (e.g., ensuring attention to unequal power dynamics and mitigation strategies were reflected in coding). In keeping with the aim of ID, which is the development of findings that have implications for and application to clinical practice contexts, we shifted to a more abstract, conceptual analysis of themes in the final stages of analysis (41, 42, 45). Evolving themes were brought to the larger team for critique and discussion as the analysis progressed, which incorporated processes for reflexivity (e.g., questioning if we were missing a particular perspective given our disciplinary orientation) and theoretical grounding (e.g., explicitly discussing how our analytic insights related to our theoretical perspectives).

### Credibility of analysis

The credibility of our analysis was supported through the use of multiple sources of data (triangulation), which supported data completeness and provided varying perspectives. An audit trail of analytic and interpretive decisions, and feedback from the broader research team was maintained throughout analysis. Finally, prior to completing our analysis, we hosted six engagement sessions to discuss study findings as a strategy to support confirmability and a way to demonstrate reciprocity to the community. We invited service providers affiliated with our partner organization (3 sessions) and community members (3 sessions) to engage in conversations about our findings and consider possible next steps for engagement and/or research.

## Findings

**Figure 1:**
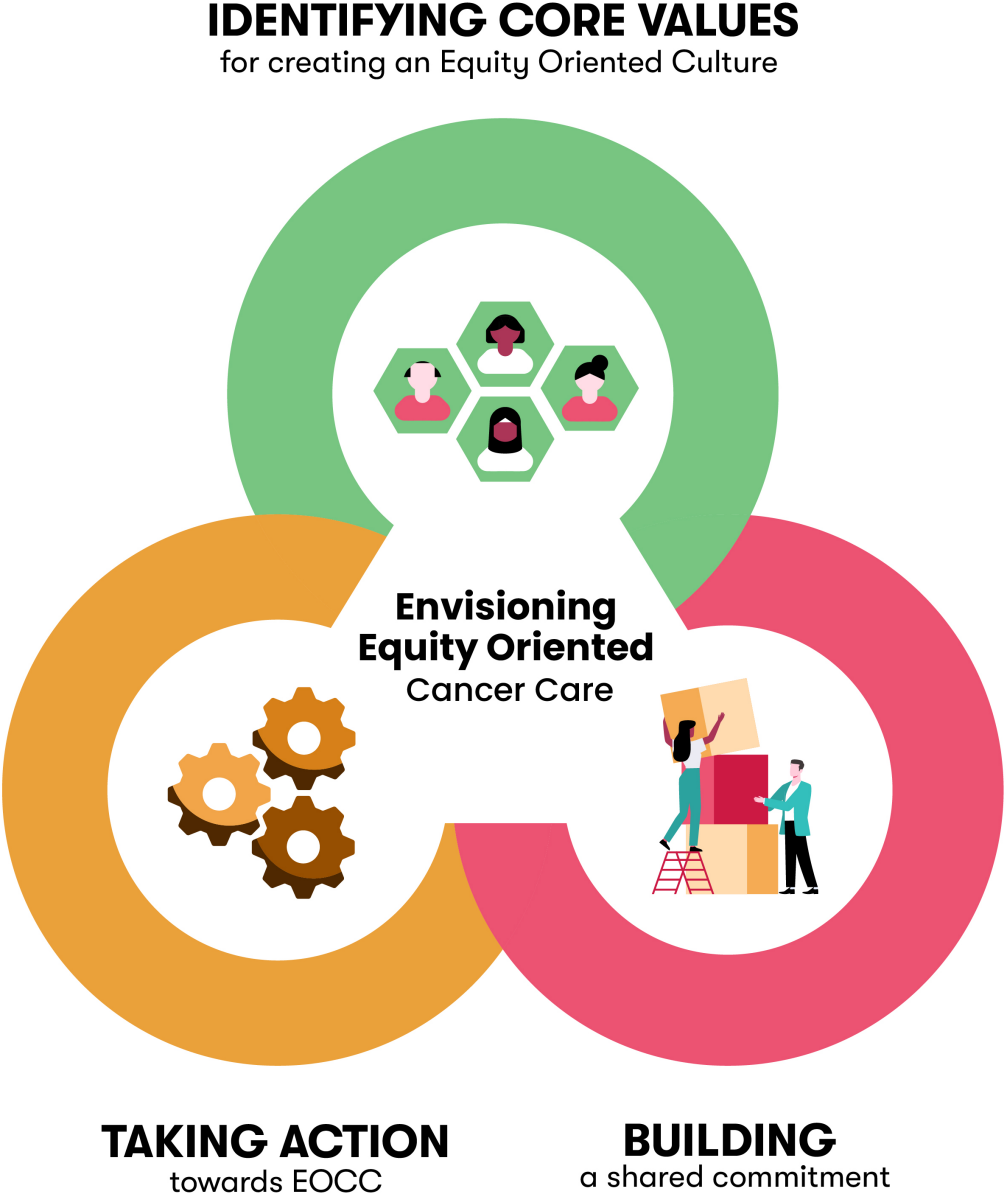
Organizational-Level Facilitators of Equity-Oriented Cancer Care

Participants were asked about how organizations delivering cancer services could make care more equitable and accessible; in response, participants provided insights and shared their experiential knowledge of equity-oriented practices. Three interconnected themes were developed that support an organizational approach to EOCC: 1) identifying core values for creating an equity-oriented culture, 2) building a shared commitment to EOCC, and 3) taking action towards EOCC (**Figure 1**).

### Theme 1: identifying core values for creating an equity-oriented culture

Values are a central to organizational culture, underlying policy decisions, guiding staff behaviours, and upholding organizational norms. Our analysis highlighted the importance of values in creating organizational contexts that support EOCC. We identified four key interrelated values that contributed to an equity-oriented culture: relationality, cultural safety, adaptability, and trust-building.

#### Relationality

Relationality can be understood as the fundamental interconnectedness and interdependence of all things; in healthcare contexts, this also conveys an understanding of the integral connections between individuals, families, communities, health systems, and sociopolitical structures (46). Drawing on the concept of relationality, participants described the importance of prioritizing strong relationships within the organization and beyond, in recognition of the interconnectedness of providers, patients, communities, and partner organizations. For example, “we really are invested … in community. And the community is not [just] the clients; the community is all of us. So, our relationship with each other is very important as it is with our clients” (SP11). Relationality was evident in how some participants described the importance of making space for collaboration and developing a conscious awareness of systems of oppression and power.

Participants felt that organizations valuing relationality could shift the organizational culture from being largely based on biomedical models of engagement and care (e.g., minimal opportunity for patient/community engagement, not taking social inequities into account), towards meaningfully integrating other ways of knowing (e.g., Indigenous teachings). Several participants advocated for the integration of other ways of knowing alongside biomedicine that take into account psychological, social and spiritual care needs:

“…the solution to this quandary isn’t going to be like, ‘What are we replacing biomedicine with?’, it will be ‘what are we adding to biomedicine so that we’re getting closer to serving the *whole* person rather than like the cells that make up their tumour?’” (KI01)

These perspectives were shared by many community member participants, who emphasizes the importance of recognizing non-biomedical ways of knowing and healing in providing equitable, person-centred care. One focus group participant remarked regarding their own use of traditional Indigenous medicine, “we need to work together … if we are going to succeed” (Focus Group 1 participant).

#### Cultural safety

Cultural safety originated in response to the inequities experienced by Indigenous peoples’ in New Zealand and has since been taken up widely in Canadian medical and nursing professional practice standards. Cultural safety recognizes and actively confronts unequal power dynamics, discrimination, and racism embedded in healthcare settings and social structures (47, 48). Cultural safety offers a framework for reforming healthcare practices, policies, and organizations by centering social justice aims, and by shifting away from locating ‘the problem’ within individual difference to examining healthcare organizations as potential “sites for transformation”. For one key informant, a shift towards cultural safety meant that within the organization, there were “…more conversations and buy-in from senior leadership … And more of an intention to actually integrate the principles of equity into all of the projects and initiatives that are happening…”. For this participant, valuing cultural safety meant the organization was “…more focused on serving the needs of the patient versus the needs of the organization…” (KI07). Another key informant spoke to the impact of actively attending to unequal power dynamics:

“…there’s an openness, there’s an inquiry around what’s most important to you. It may not be that you’re living with cancer right now or that it’s progressing. It may not be that. So what *is* most important? And to have space for that. And that it’s not just a question that I have to ask here on the form because it’s what we do but it’s actually something that is integrated. And that one can action on it as much as possible.” (KI06)

Several participants described their experiences of working within teams aligned with the aims of cultural safety: these teams were continually attuning to systems of injustice and hierarchies within the care team and the broader organization. During observations with one community-based cancer care team, attention to power and social justice was seen through how the team conveyed respect and non-judgment towards all patients, regardless of their history, circumstances, or challenges, and through their intentional and respectful use of language. For example, the team took a strengths-based approach to discussing how they could connect with a patient who had missed two weeks of prescription medications, with careful attention to using non-judgmental language. The necessity of cultural safety was underscored across all focus groups with community members, stemming from pervasive negative healthcare experiences, with one participant poignantly stating, “I’d like people to see me as human” (Focus Group 1 participant).

#### Trust-building

Analysis of our data indicated that understanding power dynamics and multiple perspectives (as in cultural safety and relationality) allowed teams to build trust with each other, and trust was seen to be foundational to advancing equity: “working in the equity space takes time … it’s predicated on strong relationships … you have to build trust” (KI05). Developing trust within and among teams facilitated agency and collaboration, allowing providers to leverage their expertise and tailor their care, a hallmark of EOCC. This was echoed by a service provider who shared: “Everyone is very collaborative, everyone knows everyone’s doing their best. Everyone trusts each other … [W]e engage in frequent collaboration in huddle and have case conferences where we discuss client goals together. So, it feels more shared” (SP23).

#### Adaptability

Participants, particularly service providers, described the necessity of adaptability, and the importance of openness and innovation among the care team to providing EOCC. For service provider participants, valuing and supporting adaptability created the workplace conditions – in primary and oncology care – to provide individualized, person-centred care. Many service providers highlighted flexibility in appointments (e.g., time of day, length, mode, ability to reschedule) as one example of adaptability ‘in action’. Our analysis revealed that the values of cultural safety, trust and relationality contributed to adaptability. For example, several service providers in community-based primary care described how adaptability allowed for innovations in care including one example of a nurse-led system to administer FIT testing (colon cancer screening), and another in which cervical self-sampling kits (cervical cancer screening) were distributed and collected by outreach workers. These examples demonstrated how adaptability facilitated creativity, but also how a relational lens allowed for a contextual understanding of the patient group they were working with (e.g., recognition of the intersecting impacts of social inequities, trauma, and life experiences that require alternative options to accessing care), how trust within teams allowed healthcare providers to take on new roles, and how the value of cultural safety led to an appreciation of power imbalances and recognition that patients should be partners in care.

### Theme 2: building a shared commitment to equity-oriented cancer care

The four interconnected core values discussed above underlie and support the capacity for organizational commitments to equity. Part of building a shared commitment to health equity is “a genuine recognition that it’s everybody’s business” (KI04). To substantiate organizational commitments, participants cited co-creating investment priorities, such as investing in resources, leadership, partnerships, and accountability mechanisms.

The most commonly discussed investment was the need for dedicated resources (e.g., time, money, personnel):

“You have to support [equity] in all kinds of ways … it needs some budget, and it needs some time, and your organization needs to invest in it, no question about that … That’s most important, right from the get-go” (KI06).

Specifically, financial resources were described as necessary to support partnerships, fund new models of care (e.g., cancer navigation) and/or specific positions (e.g., equity advisors). Key informants emphasized the importance of intentional investments to convey and actualize the commitments to equity.

Both service providers and key informants acknowledged the impact that leaders have on culture and suggested investing in leadership could drive EOCC. They felt that strengthening and fostering critical leaders (formal and informal) across all levels of the organization supports EOCC. Participants discussed the importance of leaders being self-reflexive, valuing teamwork, supporting innovation, and cultivating momentum and buy-in for a shared vision of EOCC. Leading in these ways can role model the values that create space to facilitate “a shift in mindset” (KI04). For example, one service provider participant described how their manager role-modelled adaptability by supporting providers in their department to find creative solutions to scheduling treatment appointments for a patient experiencing homelessness. One key informant described how critical leadership facilitates building organizational commitment:

“[I]t requires people look inward … to think about or reflect on their own behaviour … privilege and their positionality, and ask hard questions of themselves … [And] be bold and courageous and say, ‘yeah, some of this stuff is going to take more time. And so, I’m going to stand behind.’ But to do that you have to really understand what it is you need to do and believe that it is the right thing to do.” (KI05)

Participants suggested ongoing, mutually beneficial partnerships (e.g., with community groups, primary care organizations) could strengthen commitments for EOCC, including fostering co-development and shared accountability; creating and sustaining authentic relationships built on trust were important components of partnerships. Participants suggested that collaborative partnerships could be used to facilitate the co-design of formalized EOCC processes, as with equity-deserving communities or being open to learning from other organizations moving towards EOCC. At the same time, participants recognized that working in partnership may feel foreign or uncomfortable as organizations step back and allow for partners to lead (values of cultural safety and flexibility): “It’s working with people that are actually the most impacted and saying, what’s the biggest problem, and then developing the solution collaboratively and implementing that solution … and don’t just come to the table with a pre-baked model…” (KI03)

Participants were not always clear if their organization had formal commitments to equity written into mission, vision, values statements; strategic plans; policies; and processes and procedures. Even so, they acknowledged the need to move from mere statements to operationalizing those commitments (e.g., implementing new models of care; see theme 3b) and creating accountability mechanisms (e.g., collecting equity specific data; see theme 3a). Several key informants shared that aligning with identified health system priorities (e.g., team-based care) may add synergy or momentum, which can be advantageous for EOCC. For example, “…there’s an openness to [and] people listening and we’re trying to cultivate that [ … ] but then create some sustained actionable strategies in the work. But we couldn’t have had these conversations 10 years ago and had the same appetite for it, right?” (KI02).

### Theme 3: taking action towards EOCC

Although core values and a shared commitment are necessary steps, for EOCC to be actualized, participants highlighted the need to operationalize stated values and commitments through building accountability mechanisms and implementing tangible strategies. As one key informant outlined: “every aspect of [equity work] we do internally, across all our domains … we have a work plan and deliverables associated with that” (KI03).

#### Creating and maintaining accountability

Accountability and data are part of learning and growth systems, underpinned by core values and commitments to EOCC. These systems offer feedback loops for organizations to evaluate and adjust their efforts toward EOCC. One service provider described the importance of organizational commitment as extending into data, stating:

“[T]he organization has to be committed to it … in real ways that they’re going to change policies … They’re going to be looking at what kind of data that we’re collecting so that we can actually see what the baseline of some of the inequities … getting some better data and linking up data.” (SP20)

Participants regarded data as important for understanding existing inequities, developing interventions to address inequities, evaluating changes, and evolving according to needs. Meaningful data collection meant creating appropriate infrastructure, ensuring timeliness, and gathering meaningful input from communities impacted by health and social inequities. Participants suggested that organizations analyze data such as access to care indicators, population-level outcomes, and patient-reported outcome and experience measures, and establish data linkages (e.g., cancer registry).

Several key informants described accountability measures as necessary companions to organizational commitments: “when you don’t have data, you cannot be held accountable…” (KI05). Examples of accountability measures described by participants included individual performance plans with equity components, and patient experience surveys that included equity-related demographic information. Leaders were also seen as playing an important role in accountability structures:

“The role of leadership I think is to set the expectations of this work being done … [N]ot just put them in strategic plans that hold them accountable, but making those things accountable to the team … it’s saying to the manager at the clinic level, what is your equity-oriented indicator when you go to move to implement [a new model of care] at your site?… So, it has to go all the way down the chain from leaders to staff to say, ‘this is important, this is what we’re doing about it,’ and working with them to make it happen.” (KI07)

Implied in the above quote is also the imperative of continual evaluation of EOCC strategies to understand where strategies are effectively contributing to EOCC, and where they are not.

#### Strategies to support EOCC

Participants provided various examples of strategies, which, from their perspective, could be implemented and/or leveraged to operationalize EOCC (**Table 4**). Sample strategies are provided in **Table 4** to provide additional context to findings, and to support envisioning EOCC.

**Table 4:** Sample strategies to support EOCC.

Strategy type	Sample approaches	Illustrative quote	Potential challenges/considerations
Clinical workflows and processes to assess healthcare, social and equity needs	Initial contact and assessment upon referral receipt to identify and mitigate barriers to first appointment Integrate social and structural determinants of health assessments into clinical workflows Develop electronic chart flags that clearly identify unique equity needs	“Have well described workflows, first of all, to screen for people who have particular needs and then algorithms that staff in different disciplines, clerks and admitting and so on, can follow so that those patients get the care that they need.” (KI04) “So it would be good if the organization … flags people up front for having those needs and then could book those patients in for a length of time. But also at a time of day that is good for both the patient and the provider [ … ] all of their potential needs should be identified up front in the consultation … basically people screened for lower socioeconomic status, for health literacy.” (SP20)	Risk of creating a ‘checklist’ approach to assessments of social and structural determinants of health Assessments of determinants of health, including social needs, should consider what information is essential for understanding patient barriers and avoid unnecessary/potentially stigmatizing questions Need for staff training on sensitive conversations Risk of perpetuating stigma with use of chart flags if not paired with staff education and non-stigmatizing language
Multi-disciplinary and intersectoral collaboration to support access to cancer services	Pre-admission to hospital, reserved beds, or temporary supportive housing units to provide shelter for people requiring cancer screening (e.g., bowel preparation and colonoscopy) or treatment Collaborate with homecare or outreach teams in primary care to facilitate patient communication, care coordination, and access to screening services	“Our social work team will find the funds to put them in a hotel, that happens actually quite frequently … Often, it’s an offering, to stay in our cancer lodge, which is just across the road where they get three meals a day. Sometimes they get a private, but maybe a shared accommodation. And then they can walk back and forth for their treatments…” (SP17) “Our outreach capabilities as a team have really facilitated access in that we can give folks screening devices or diagnostic tests, like FIT test or HPV tests, then they can do them at home. And then we can send outreach workers to collect them.” (SP23)	Collaboration with external organizations may be impeded as a result of legal frameworks (e.g., privacy laws) Relationships that are dependent upon individuals rather than organization to organization or more formal relationships may not be sustainable (i.e., the ‘collaboration’ ends when the individual leaves their role)
Organizational approaches to cancer care delivery to address health and social care needs	Implement integrated or collaborative team-based care models to address a range of care needs (e.g., oncologist, nurse, social worker, medical assistant, Elder) Implement care coordination models such as case management or nurse navigation for patients with a higher level of support need. These may be supported by multi-disciplinary case conferences or rounds that emphasize social needs Develop resource and referral pathways to address unmet social and structural needs identified during assessment	“I have seen – it appears to me that like the whole [cancer institution] machinery has enveloped [patients experiencing major barriers to care] in a positive way and just has sort of taken that on and they’re getting wraparound care from [cancer institution] … what I mean by wraparound, is the teams are communicating effectively. There’s lots of support people that are facilitating the different treatments and appointments and whatnot.” (SP02) “A case management model of care, or a nurse navigator model of care, where there is someone tasked with coordinating various aspects of care, and communicating between different care providers. In addition this could provide a consistent ‘point person’ for the patient so that trust could be built and there was a sense of caring and security.” (SP19)	Collaborative and/or team based care models must go beyond simply placing multi-disciplinary HCP in roles. Attending to power dynamics/hierarchies, developing care processes, communication mechanisms, and role clarity are essential Cancer service delivery organizations may not have sufficient resources to manage an influx of referrals for unmet social/structural needs Participating in multi-disciplinary rounds may be viewed as taking away from clinical time; consideration should be given to building this into clinical workflows or role descriptions
Patient and family supports embedded within cancer service delivery organizations	Provide cultural and spiritual supports (e.g., spiritual care, Elders and knowledge keepers, traditional medicine) Provide patient representatives or personal support workers to accompany patients to appointments and provide individualized support (e.g., ‘cancer doula’) Provide logistical supports (e.g., taxi vouchers, volunteer drivers to support transportation needs)	“…bringing their culture in, no matter what it is, whether it be Aboriginal, whether it be Jewish, whatever it is, if they need a Rabbi, to be with them, or an Elder to be with them, bring that in, makes a huge difference.” (SP10)	Staff may lack competency in identifying when such supports are needed Consideration is needed as to how such supports could be integrated into a team-based approach rather than an arms-length provider
Physical spaces and equity-oriented environments	Create welcoming physical spaces (e.g., using artwork) Provide comfort resources in waiting rooms (e.g., food/water, harm reduction supplies and spaces) Create harm reduction policies	“An episodic overdose prevention service where a space is made available for someone to use if they need to, while they’re waiting” (SP02)	Limited budgets and/or support for changes perceived as being ‘cosmetic’ Some changes that make physical spaces feel more welcoming may not align with the realities of clinical care delivery (e.g., sparsely decorated spaces with easy to clean surfaces for infection control purposes)

### Values, commitment and action as dynamic and interconnected

Although we have presented three distinct themes in our analysis, we emphasize that these dimensions (values, commitments and actions) are dynamic and mutually reinforcing. One key informant described this interaction as: “there’s no short way to advance health equity. This is a multi-pronged, multi-sectoral, multi-layer approach that we need to take” (KI05). For example, equity-oriented values will inform organizational commitments and may reciprocally be demonstrated through those commitments. Organizational commitments to EOCC may also reveal the need for exploration and co-creation of values to underpin those commitments. Similarly, accountability structures and evaluation of tangible strategies (taking action) will aid in understanding which strategies are effective, but may also serve to identify gaps in strategic goals or resources (commitments); data (taking action) may be used to generate greater buy-in and commitments. Finally, accountability structures (taking action) including reporting on equity indicators to equity-deserving communities may be informed by values such as relationality.

## Discussion

We examined organizational facilitators of equity-oriented care through observations and conversations with cancer care stakeholders. While most of the literature thus far discusses EOCC at the healthcare provider level and often situates EOCC within the language of person-centred care, our study offers insights regarding how cancer service delivery organizations can create contexts that enhance capacity for EOCC. Although, to our knowledge, this is the first study focusing on organizational facilitators of EOCC, our analysis complements existing frameworks to support equity-oriented care and health equity actions, including approaches to organizational values work and equity-oriented leadership styles, while recognizing equitable care as a key component of quality care (48, 49). For example, research focusing on organizational facilitators of equity-oriented care in primary care and emergency department settings highlights how meaningful engagement, attending to power differentials, actively countering discrimination, and revisioning use of time are foundational to supporting equity-oriented care (16, 18–22). Similarly, we found that relationality, cultural safety and adaptability (respectively) were foundational values supporting EOCC. Research demonstrates that investing in leadership, trust-building amongst teams, and partnerships are core components of well-functioning organizations (50–52); our findings indicate that these also support an equity-oriented approach.

Findings from this study emphasize the importance of acting at the organizational level to lay a foundation for EOCC, and highlight how embedding equity into organizational culture can facilitate EOCC at the patient level (53). However, although equity may be part of some organizations’ stated values, a lack of conceptual clarity on how equity is understood can leave organizations struggling to make meaningful strides towards realizing equity (14, 54). To enhance and foreground an organizational culture of equity and foster tangible equity-oriented actions (53), we proposed four overlapping values of relationality, cultural safety, adaptability, and trust-building as laying the foundation for organizations to facilitate EOCC. While many frameworks offer evidence-informed strategies to promote equity (16, 51, 53, 55, 56), taking a step back to first explore the values necessary to enact EOCC opens new opportunities to use those values to guide the development and implementation of EOCC that is locally relevant and tailored to context. Moreover, attending to organizational values as a *first step* may provide an anchor for equity work in addition to sustaining momentum in an environment where there are competing demands and shifting priorities.

However, as administrators and leaders in the cancer care sector consider the applicability of these values to their specific organizational context, they may also want to consider their organizational ‘operating system’. Scholars in the field of organizational leadership discuss relational *ego*systems and relational *eco*systems as two ways that organizations function: *Ego*systems are based on individuality and independence whereas *eco*systems position individuals as part of an interdependent, connected collective (50). Our findings support the importance of building *eco*systems where organizational cultures grounded in high-quality, reciprocal, trusting relationships lay a foundation for creating a culture of equity (50, 53). This is in tension with dominance of individualism and acute care culture which is well documented to pervade western biomedical healthcare systems and preclude equity-oriented approaches (15, 26, 57, 58). Organizations aiming to shift to EOCC might consider adopting the core values we have outlined as *core conditions* foundational to support organizational change. In contrast, organizations that misappropriate the values outlined or attempt to prescribe values through a top-down approach risk resistance to change and a misaligned organizational culture (50), but more concerningly, risk implementing performative strategies (e.g., checklist approaches) that may perpetuate harm rather than meaningfully advance health equity. Specifically, research suggests that traditional power imbalances, hierarchical organizational cultures and professional silos can present significant challenges to organizational change supporting equity-oriented approaches (53, 59). However, inviting staff to be actively involved in ongoing and evolving conversations about embedding equity within the organization supports success in adapting to and sustaining change (60, 61). Some scholars have suggested that a process of values inquiry and open dialoguing invites curiosity, discovery, and relationality (62). Given how organizations are fraught with power dynamics, a decentralized approach to co-creating values within an organization can cultivate a relational ecosystem. This requires ongoing shifting of power (53, 60), non-hierarchical and engaged discussions (50, 63), and curiosity and openness to the evolving nature of co-creation (64), which, in turn, enhance staff engagement, motivation, and support rewarding workplaces (63). Other examples of operationalizing equity within the organization, such as through ongoing and progressive training for all staff, have been shown to promote greater buy-in, ownership, and sustainability of change toward collective goals (55, 60, 61). These contribute to relational ecosystems where job satisfaction, group resilience, and teamwork are enhanced, and the delivery of EOCC is supported (16, 65).

Leadership is a cornerstone of promoting relational ecosystems, supporting organizational change, and advancing equity (49–52, 56, 63, 66, 67). As a key investment in our framework, we highlight the importance of both formal and informal leadership across organizations (68); moreover, *how* leaders lead is critical (67). Leaders who employ critical leadership (64), transformational leadership (63), or coordinated relational approaches (high-quality connection and communication) (50) can facilitate advancements toward EOCC. Critical leadership in particular requires leaders to understand social and structural determinants of health, develop power literacy, and gain capacity for complex adaptive systems thinking (64), all essential to integrating EOCC. A critical leadership style and relational coordination approach can enable leaders to embody equity-oriented connections and communication, building meaningful, reciprocal partnerships with the community and other stakeholders (16, 50, 64). Decentralized approaches to decision-making, and anticipating resistance and conflict are also important in generating buy-in as organizations take meaningful steps toward equity (16, 27, 60). Organizations also play a role in supporting leaders through meaningful training (16, 27, 55) as well as supporting their engagement in and resources for equity transformation. For instance, leaders who are supported to change policy and processes in turn bolster healthcare providers’ ability to work in equity-oriented ways and improve patient care delivery of EOHC (60, 65, 69). Yet significant challenges remain to educating, recruiting and maintaining such leaders in healthcare systems that favour operational oversight, system efficiencies, and standardized care over critical or transformational leadership (69). Moreover, even in the presence of skilled leadership, organizations may encounter resistance to advancing health equity related to the broader health systems context, including competing policy directives (e.g., promoting health equity and delivering person-centred care vs. maintaining system efficiency and maximizing productivity), and health human resources challenges (e.g., healthcare provider burnout and attrition post pandemic) (10). We have more fully explored the challenges to integrating EOCC elsewhere (10).

A critical lever in advancing health equity includes embedding mechanisms for accountability; these can also support strategic initiatives, including service and program planning and evaluation, tailored to the specific equity needs of patients and populations (26, 49, 70). For example, in prior research on the effectiveness of equity-oriented interventions in primary care organizations, implementation of interventions was significantly enhanced by using data on patient and population health to focus on specific interventions to promote EOHC (16, 18, 19, 22). At the same time, caution must be exercised when selecting and using measurement tools given the immense gaps and challenges of appropriate data collection and assessment of patient and community equity needs (14, 51, 53). Meaningfully engaging communities, from project conceptualization through to dissemination and use of data is vital in the pursuit of EOCC (71). We echo the Canadian Cancer Society and Canadian Partnership Against Cancer (70) who advocate for the development of partnerships (e.g., cross-jurisdictional and cross-sectoral; partnerships with community, patient, family and caregivers) as crucial to identifying locally-relevant and appropriate equity indicators and mobilizing data to support EOCC.

In her examination of critical leadership, McGibbon (64) notes that being *for* equity is more than being *against* discrimination, implying that work to advance health equity must be planned, purposeful and forward-moving. As such, EOCC cannot be reduced to a checklist or collection of written statements. Instilling change at the values level is a crucial step, but enacting EOCC also requires formalizing commitments and actualizing them through policies, processes, and procedures. These intentional and tangible commitments may help realize equity-oriented values and move organizations away from reactionary, temporary and/or ‘band-aid’ solutions. Shifting to relational models of leadership (as described above) can support a more proactive approach to healthcare planning, which, when combined with meaningful data points, can be used implement equity-oriented care strategies (16, 63, 72). In **Table 4**, we offer some strategies to support taking concrete action toward EOCC, derived from our data; a fulsome discussion of recommendations to enhance organizational capacity for EOCC will be presented in a forthcoming paper. However, we emphasize that efforts to advance EOCC will require thoughtful planning, robust engagement with stakeholders, and strategic investments at multiple levels.

## Strengths and limitations

While we have provided a general framework for advancing EOCC from the organizational level, it is important to acknowledge that this research was conducted within a specific context—focusing on one province and two large urban areas. Further research is needed to explore organizational facilitators of EOCC more broadly. Moreover, research to explore effective implementation of EOCC is needed, as implementation of equity-oriented approaches is complex, and highly context dependent (22). Our approach to inviting community member perspectives (i.e., invitations through our community partner organization) may have resulted in over-representation of service-connected community members; however, given the aims of the study, community members perspectives were highly informative and served as an entry point to deepen our understanding. Through continued engagement and relationship building with these communities, we hope to be able to broaden our engagement with those less connected to services in the future. Nonetheless, our study’s strengths include its methodological rigour, particularly the triangulation of multiple data sources and types, and its contribution to the expanding body of literature on health equity and cancer care.

## Conclusion

Advancing equity in cancer care and addressing entrenched inequitable cancer outcomes among structurally marginalized populations will require action at multiple levels. Organizations delivering cancer services are increasingly recognized as sites of transformation, with the potential to reduce barriers to accessing cancer care and mitigate the impacts of social and structural determinants of health through equity-oriented approaches to care and health equity actions. Although EOHC is one approach thought to hold promise, little research has explored the delivery of EOHC within the cancer care context (as in EOCC) and the organizational factors that support such an approach. Our findings highlight the important role of shared organizational values and commitments to equity. In particular, the co-creation of values and commitments has the potential to generate the contextual conditions needed to support EOCC. Yet values and commitment will be ineffective if not paired with tangible actions and meaningful accountability measures. Our findings offer a starting place for organizations delivering cancer services who want to embed equity into their approach to care.

## Data Availability

The datasets presented in this article are not readily available because of privacy and ethical considerations. Requests to access the datasets should be directed to Tara Horrill, tara.horrill@umanitoba.ca.

## Footnotes

1 Structural marginalization refers to the process of marginalization and exclusion resulting from political, economic, social and historical structures, and the uneven distribution of resources, power and opportunities. Structural marginalization shifts away from an understanding marginalization as a result of individual characteristics to a focus on the impacts of social structures (23).

## References

[1] Canadian Partnership Against Cancer . Examining disparities in cancer control system performance special focus report. (2014), 88–.

[2] Asgary R . Cancer screening in the homeless population. Lancet Oncol. (2018) 19:e344–50. doi: 10.1016/S1470-2045(18)30200-6. PMID: 30084381

[3] Davis LE Coburn NG Hallet J Earle CC Liu Y Myrehaug S . Material deprivation and access to cancer care in a universal health care system. Cancer. (2020) 126:4545–52. doi: 10.1002/cncr.33107. PMID: 32745271

[4] Mahar AL Kurdyak P Hanna TP Coburn NG Groome PA . The effect of a severe psychiatric illness on colorectal cancer treatment and survival: A population-based retrospective cohort study. PloS One. (2020) 15:e0235409–e0235409. doi: 10.1371/journal.pone.0235409. PMID: 32726314 PMC7390537

[5] Booth CM Li G Zhang-Salomons J Mackillop WJ . The impact of socioeconomic status on stage of cancer at diagnosis and survival: A population-based study in Ontario, Canada. Cancer. (2010) 116:4160–7. doi: 10.1002/cncr.25427. PMID: 20681012

[6] Bourgeois A Horrill TC Mollison A Stajduhar KI . Barriers to cancer treatment and care for people experiencing structural vulnerability: A secondary analysis of ethnographic data. Int J For Equity Health. (2023) 22:e58. doi: 10.1186/s12939-023-01860-3. PMID: 36998035 PMC10064679

[7] Bourgeois A Horrill T Mollison A Stringer E Lambert LK Stajduhar K . Barriers to cancer treatment for people experiencing socioeconomic disadvantage in high-income countries: a scoping review. BMC Health Serv Res. (2024) 24:670. doi: 10.1186/s12913-024-11129-2. PMID: 38807237 PMC11134650

[8] Horrill TC Martin DE Lavoie JG Schultz ASH . A critical exploration of nurses’ perceptions of access to oncology care among Indigenous peoples: Results of a national survey. Nurs Inq. (2021) 29s(1):e446. doi: 10.1111/nin.12446. PMID: 34342080 PMC9286560

[9] Horrill TC Martin DE Lavoie JG Schultz ASH . Access denied: Nurses’ perspectives of access to oncology care among Indigenous peoples in Canada. Adv Nurs Sci. (2022) 45:292–308. doi: 10.1097/ANS.0000000000000428. PMID: 35696372

[10] Horrill TC Crawford J Beck SM Bourgeois A Kaur J Lambert L . There’s just such a mismatch”: A qualitative exploration of health systems and organizational-level barriers to accessing cancer services among people experiencing structural marginalization. Int J For Equity Health. (2025) 24:181. doi: 10.1186/s12939-025-02554-8. PMID: 40533755 PMC12177988

[11] Sutcliffe SB . A review of Canadian health care and cancer care systems. Cancer. (2011) 117:2241–4. doi: 10.1002/cncr.26053. PMID: 21523740

[12] Baah FO Teitelman AM Riegel B . Marginalization: Conceptualizing patient vulnerabilities in the framework of social determinants of health—An integrative review. Nurs Inq. (2019) 26:e12268. doi: 10.1111/nin.12268. PMID: 30488635 PMC6342665

[13] Alcaraz KI Wiedt TL Daniels EC Yabroff KR Guerra CE Wender RC . Understanding and addressing social determinants to advance cancer health equity in the United States: A blueprint for practice, research, and policy. CA: A Cancer J For Clin. (2020) 70:31–46. doi: 10.3322/caac.21586. PMID: 31661164

[14] Lambert LK Horrill TC Beck SM Bourgeois A Browne AJ Cheng S . Health and healthcare equity within the Canadian cancer care sector: a rapid scoping review. Int J Equity Health. (2023) 22:20. doi: 10.1186/s12939-023-01829-2. PMID: 36709295 PMC9883825

[15] Horrill TC Browne AJ Stajduhar KI . Equity-oriented healthcare: What it is and why we need it in oncology. Curr Oncol. (2022) 29:186–92. doi: 10.3390/curroncol29010018. PMID: 35049692 PMC8774995

[16] Browne AJ Varcoe C Ford-Gilboe M Nadine Wathen C Smye V Jackson BE . Disruption as opportunity: Impacts of an organizational health equity intervention in primary care clinics. Int J For Equity Health. (2018) 17:1–16. doi: 10.1186/s12939-018-0820-2. PMID: 30261924 PMC6161402

[17] Wyatt R Laderman M Botwinick L Mate K Whittington J . Achieving health equity: A guide for health care organizations. Cambridge, Massachusetts: Institute for Healthcare Improvement (2016). Report No.

[18] Browne AJ Varcoe CM . EQUIP Healthcare: An overview of a multi-component intervention to enhance equity-oriented care in primary health settings. Int J For Equity Health. (2015) 14:152. doi: 10.1186/s12939-015-0271-y. PMID: 26694168 PMC4688920

[19] Browne AJ Varcoe C Ford-Gilboe M Wathen CN Wilson E Bungay V . Using a health equity lens to measure patient experiences of care in diverse health care settings. PloS One. (2024) 19:e0297721–e0297721. doi: 10.1371/journal.pone.0297721. PMID: 38843218 PMC11156339

[20] Varcoe C Browne AJ Bungay V Perrin N Wilson E Wathen CN . Through an equity lens: Illuminating the relationships among social inequities, stigma and discrimination, and patient experiences of emergency health care. BMC Health Serv Res. (2022) 52:246–60. doi: 10.1177/00207314221075515. PMID: 35098791 PMC8894974

[21] Varcoe C Browne AJ Perrin N Wilson E Bungay V Byres D . EQUIP emergency: can interventions to reduce racism, discrimination and stigma in EDs improve outcomes? BMC Health Serv Res. (2022) 22:NA–A. doi: 10.1186/s12913-022-08475-4. PMID: 36050677 PMC9436447

[22] Browne AJ Varcoe CM . Taking action at the organizational level: Creating a context for implementing trauma- and violence-informed care in health care and other sectors. In: Implementing trauma- and violence-informed care: A handbook. Toronto, Canada: University of Toronto Press (2023). p. 85–98.

[23] Powell JA . Deepening our understanding of structural marginalization. Poverty Race. (2013) 22:3–5.

[24] Davidson CA Kennedy K Jackson KT . Trauma-informed approaches in the context of cancer care in Canada and the United States: A scoping review. Trauma Violence Abuse. (2023) 24:2983–96. doi: 10.1177/15248380221120836. PMID: 36086877 PMC10594848

[25] Cunningham J Rumbold AR Zhang X Condon JR . Incidence, aetiology, and outcomes of cancer in Indigenous peoples in Australia. Lancet Oncol. (2008) 9:585–95. doi: 10.1016/s1470-2045(08)70150-5. PMID: 18510990

[26] Cohen BE Schultz A McGibbon E VanderPlaat M Bassett R GermAnn K . A conceptual framework of organizational capacity for public health equity action (OC-PHEA). Can J Public Health. (2013) 104:e262–266. 10.17269/cjph.104.3735 PMC6973720 23823893

[27] Spitzer-Shohat S Chin MH . The “Waze” of inequity reduction frameworks for organizations: a scoping review. J Gen Intern Med. (2019) 34:604–17. doi: 10.1007/s11606-019-04829-7. PMID: 30734188 PMC6445916

[28] O’Brien BC Harris IB Beckman TJ Reed DA Cook DA . Standards for reporting qualitative research: A synthesis of recommendations. Acad Med. (2014) 89:1245–51. doi: 10.1097/ACM.0000000000000388. PMID: 24979285

[29] Tong A Sainsbury P Craig J . Consolidated criteria for reporting qualitative research (COREQ): a 32-item checklist for interviews and focus groups. Int J Qual Health Care. (2007) 19:349–57. doi: 10.1093/intqhc/mzm042. PMID: 17872937

[30] Madison DS . Critical ethnography: method, ethics, and performance. Los Angeles, CA: SAGE Publications Inc (2005).

[31] Thomas J . Doing critical ethnography. Los Angeles, CA: SAGE Publications Inc (1993).

[32] Kothari A Wathen CN . Integrated knowledge translation: Digging deeper, moving forward. J Epidemiol Community Health. (2017) 71:619–23. doi: 10.1136/jech-2016-208490. PMID: 28298415

[33] Kothari A Wathen CN . A critical second look at integrated knowledge translation. Health Policy. (2013) 109:187–91. doi: 10.1016/j.healthpol.2012.11.004. PMID: 23228520

[34] Reimer Kirkham S Browne AJ . Toward a critical theoretical interpretation of social justice discourses in nursing. Adv Nurs Sci. (2006) 29:324–39. doi: 10.1097/00012272-200610000-00006. PMID: 17135801

[35] Anderson JM Rodney P Reimer-Kirkham S Browne AJ Khan KB Lynam MJ . Inequities in health and healthcare viewed through the ethical lens of critical social justice: Contextual knowledge for the global priorities ahead. Adv Nurs Sci. (2009) 32:282–94. doi: 10.1097/ANS.0b013e3181bd6955. PMID: 19934835

[36] Kincheloe JL McLaren P . Rethinking critical theory and qualitative research. 3rd ed. Denzin NK Lincoln YS , editors. Thousand Oaks, CA: Sage Publications (2005) p. 303–38.

[37] Strega S . The view from the poststructural margins: Epistemology and methodology reconsidered. 2nd ed. Strega S Brown L , editors. Toronto, Canada: Canadian Scholars’ Press (2015) p. 119–52.

[38] Dhamoon RK Hankivsky O . Why the theory and practice of intersectionality matter to health research and policy. In: Hankivsky O , editor.Health inequities in Canada: Intersectional frameworks and practices. UBC Press, Vancouver, Canada (2011).

[39] Hankivsky O Christoffersen A . Intersectionality and the determinants of health: A Canadian perspective. Crit Public Health. (2008) 18:271–83. doi: 10.1080/09581590802294296. PMID: 41909888

[40] Madison DS . Critical ethnography: method, ethics, and performance. Los Angeles, CA: SAGE Publications Inc (2005).

[41] Thorne S . Interpretive description: Qualitative research for applied practice. 2nd ed. New York, NY: Routledge (2016).

[42] McCall J Phillips JC Estefan A Caine V . The relationship between critical social theory and interpretive description in nursing research. Global Qual Nurs Res. (2023) 10:23333936231211462. doi: 10.1177/23333936231211462. PMID: 38028738 PMC10676628

[43] Horrill TC Varcoe C Brown H Stajduhar KI Browne AJ . Bringing an equity lens to participant observation in critical ethnographic health research. Int J Qual Methods. (2024) 23:16094069241270397. doi: 10.1177/16094069241270397. PMID: 41914793

[44] Thorne S Reimer-Kirkham S O’Flynn-Magee K . The analytic challenge in interpretive description. Int J Qual Methods. (2004) 3:1–7. doi: 10.1177/160940690400300101. PMID: 41914793

[45] Thorne S Stephens J Truant T . Building qualitative study design using nursing’s disciplinary epistemology. J Advanced Nurs. (2016) 72:451–60. doi: 10.1111/jan.12822. PMID: 26412414

[46] Hartrick Doane G Varcoe C . How to nurse: Relational inquiry with individuals and families in changing health and health care contexts. Philadelphia, PA: Wolters Kluwer Health (2015).

[47] Papps E Ramsden I . Cultural safety in nursing: the New Zealand experience. Int J Qual Health Care. (1996) 8:491–7. doi: 10.1093/intqhc/8.5.491. PMID: 9117203

[48] Browne AJ Varcoe C Lavoie J Smye V Wong ST Krause M . Enhancing health care equity with Indigenous populations: evidence-based strategies from an ethnographic study. BMC Health Serv Res. (2016) 16:544. doi: 10.1186/s12913-016-1707-9. PMID: 27716261 PMC5050637

[49] Dzau VJ Mate K O’Kane M . Equity and quality—Improving health care delivery requires both. JAMA. (2022) 327:519. doi: 10.1001/jama.2022.0283. PMID: 35060998

[50] Cunha MPE Rego A Simpson AV Clegg S . Positive Organizational Behaviour: A reflective approach. London, UK: Taylor & Francis Group (2020).

[51] Doherty JA Johnson M McPheron H . Advancing health equity through organizational change: Perspectives from health care leaders. Health Care Manage Rev. (2022) 47:263–70. doi: 10.1097/HMR.0000000000000326. PMID: 34456273 PMC9162074

[52] Nkrumah J Abekah-Nkrumah G . Facilitators and barriers of patient-centered care at the organizational-level: a study of three district hospitals in the central region of Ghana. BMC Health Serv Res. (2019) 19:900. doi: 10.1186/s12913-019-4748-z. PMID: 31775809 PMC6882059

[53] Todic´ J Cook SC Spitzer-Shohat S Williams JS Battle BA Jackson J . Critical theory, culture change, and achieving health equity in health care settings. Acad Med. (2022) 97:977–88. doi: 10.1097/ACM.0000000000004680. PMID: 35353723 PMC9232289

[54] Amri MM Jessiman-Perreault G Siddiqi A O’Campo P Enright T Di Ruggiero E . Scoping review of the World Health Organization’s underlying equity discourses: apparent ambiguities, inadequacy, and contradictions. Int J Equity Health. (2021) 20:70. doi: 10.1186/s12939-021-01400-x. PMID: 33658033 PMC7931570

[55] Aysola J Murdock HM Lett E Williams C Wade R Higginbotham EJ . Operationalizing inclusion: moving from an elusive goal to strategic action. Epidemiologic Rev. (2023) 45:140–5. doi: 10.1093/epirev/mxad005. PMID: 37259471

[56] Vleminckx S Van Bogaert P De Meulenaere K Willem L Haegdorens F . Factors influencing the formation of balanced care teams: the organisation, performance, and perception of nursing care teams and the link with patient outcomes: a systematic scoping review. BMC Health Serv Res. (2024) 24:1129. doi: 10.1186/s12913-024-11625-5. PMID: 39334182 PMC11429156

[57] Raphael D Curry-Stevens A Bryant T . Barriers to addressing the social determinants of health: Insights from the Canadian experience. Health Policy. (2008) 88:222–35. doi: 10.1016/j.healthpol.2008.03.015. PMID: 18471923

[58] Truant TLO Lambert LK Thorne S . Barriers to equity in cancer survivorship care: Perspectives of cancer survivors and system stakeholders. Global Qual Nurs Res. (2021) 8. doi: 10.1177/23333936211006703. PMID: 33912623 PMC8050754

[59] Clack L Smith J Charns M . Defining and measuring organizational transformation in health care: A systematic literature review. Med Care Res Rev. (2026) 83:71–102. doi: 10.1177/10775587251356130. PMID: 40801357 PMC12946223

[60] Wathen N Varcoe C . Implementing trauma- and violence-informed care: A handbook. Toronto, Canada: University of Toronto Press (2023).

[61] Shen H Li C Yeh SJ . Do hospitals attaining a public recognition for treating nurses fairly deliver better‐quality health care? Evidence from cross‐sectional analysis of California hospitals. J Advanced Nurs. (2024) 80:4103–12. doi: 10.1111/jan.16123. PMID: 38382902

[62] Espedal G Carlsen A . Value inquiry and constructing the good in organizations. Organ Stud. (2024) 45:1075–98. doi: 10.1177/01708406241253161. PMID: 41914793

[63] Nurmeksela A Zedreck Gonzalez JF Kinnunen J Kvist T . Components of the Magnet® model provide structure for the future vision of nurse managers’ work: A qualitative perspective of nurse managers. J Nurs Manag. (2021) 29:2028–36. doi: 10.1111/jonm.13337. PMID: 33843122

[64] McGibbon E . Applying critical leadership to advance 2SLGBTQIA+ health equity: A complex adaptive systems approach. Healthc Manage Forum. (2023) 37:1–8. doi: 10.1177/08404704231210868. PMID: 37982709

[65] Ford‐Gilboe M Wathen CN Varcoe C Herbert C Jackson BE Lavoie JG . How equity‐oriented health care affects health: Key mechanisms and implications for primary health care practice and policy. Milbank Q. (2018) 96:635–71. doi: 10.1111/1468-0009.12349. PMID: 30350420 PMC6287068

[66] Burnes B Jackson P . Success and failure in organizational change: An exploration of the role of values. J Change Manage. (2011) 11:133–62. doi: 10.1080/14697017.2010.524655. PMID: 41909888

[67] Hogan TH O’Rourke BP Weeks E Silvera GA Choi S . Top-level leaders and implementation strategies to support organizational diversity, equity, inclusion, and belonging (DEIB) interventions: a qualitative study of top-level DEIB leaders in healthcare organizations. Implementation Sci. (2023) 18:59. doi: 10.1186/s13012-023-01319-7. PMID: 37936190 PMC10631201

[68] Espedal G Carlsen A . Don’t pass them by: Figuring the sacred in organizational values work. J Bus Ethics. (2021) 169:767–84. doi: 10.1007/s10551-019-04266-w. PMID: 41913934

[69] Thorne S . Slow death by policy manual. Nurs Inq. (2021) 28:e12442. doi: 10.1111/nin.12442. PMID: 34310823

[70] Canadian Cancer Society Canadian Partnership Against Cancer . Pan-canadian cancer data strategy (2024). Available online at: https://s22457.pcdn.co/wp-content/uploads/2023/07/Pan-Canadian-Cancer-Data-Strategy_En_Final.pdf. Report No. (Accessed June 15, 2025).

[71] Konczi AE Bill L . Advancing first nations principles of OCAP®. In: Garvey G , editor.Indigenous and tribal peoples and cancer. Springer Nature Switzerland, Cham (2024). p. 37–9. Internet. doi: 10.1007/978-3-031-56806-0_8, PMID:

[72] Sayani A . Health equity in national cancer control plans: An analysis of the Ontario Cancer Plan. Int J Health Policy Manag. (2019) 8:550–6. doi: 10.15171/ijhpm.2019.40. PMID: 31657177 PMC6815982

